# Supplementary material for: The First Whole Genome Sequencing of Agaricus bitorquis and Its Metabolite Profiling
Source: J Fungi (Basel). 2023 Apr 18;9(4):485. doi: 10.3390/jof9040485 (PMC10142948; doi:10.3390/jof9040485)
Supplement: Supplementary file 1 [file jof-09-00485-s001.zip › jof-2334904-supplementary.pdf]

The First Whole Genome Sequencing of *Agaricus bitorquis* and its Metabolite Profiling

Chunhua Zhao [1], Xi-long Feng [2], Zhen-xin Wang [2] and Jianzhao Qi [2,*]

[1] Hubei Key Laboratory of Natural Medicinal Chemistry and Resource Evaluation, School of Pharmacy, Tongji Medical College, Huazhong University of Science and Technology, Wuhan, Hubei 430030, China
[2] Shaanxi Key Laboratory of Natural Products & Chemical Biology, College of Chemistry & Pharmacy, Northwest A&F University, Yangling, Xianyang 712100, China
* Correspondence: qjz@nwafu.edu.cn; Tel: +86-029-87092381

Contents

Table S1. Statistics of Oxford Nanopore PromethION sequencing data volume of *A. bitorquis* BH01 genome. 1
Table S2. Statistics of Illumina NovaSeq sequencing data volume information of *A. bitorquis* BH01 genome. 2
Table S3. Assembly results statistics of *A. bitorquis* BH01 genome....3
Table S4. Genome size estimates for *A. bitorquis* BH01 genome. 4
Table S5. Statistics of Illumina NovaSeq sequencing data mapping of *A. bitorquis* BH01 genome. 5
Table S6. Statistics of BUSCO evaluation of *A. bitorquis* BH01 genome....6
Table S7. Annotation Statistics of coding gene from the *A. bitorquis* BH01 genome. 7
Table S8. Statistics of non-coding RNA annotation results in *A. bitorquis* BH01 genome. 8
Table S9. Statistics of *A. bitorquis* BH01 repetitive sequence annotation results....9
Table S10. Statistics of *A. bitorquis* BH01 protein-coding gene annotation....10
Table S11. The composition of CAZymes of *Agaricus* and edible mushrooms....11
Table S12. GC contents of the mitogenomes of *A. bisporus* var. *bisporus* H97 and *A. bitorquis* BH01. 12
Table S13. Analysis of orthologous genes unique to *A. bitorquis* BH01. 13
Table S14. Homologous genes of *A. bisporus* fruiting body development related genes in *A. bitorquis* BH01. 20
Figure S1. ITS alignment of the strain BH01....21
Figure S2. *K*-mer-Depth and *K*-mer Species-Frequency Distribution Plot. 22
Figure S3. Species distribution map of Nr database alignment to sequences. 23
Figure S4. Statistical map of functional annotation classification based on GO database 24
Figure S5. Statistical Chart of COG Functional Annotated Classification....25
Figure S6. KEGG Pathway Functional Classification Chart. 26
Figure S7. Domain annotation based on the Pfam database. 27
Figure S8. P450s Cluster analysis of *A. bitorquis* BH01 and other Basidiomycetes. 28
Figure S9. Molecular network analysis of metabolites from the fruiting bodies of *A. bitorquis* and *A. bisporus*. 29
Figure S10. The LC-ESI-HRMS and LC-ESI-HRMS/MS spectrums of isolates from the fruiting bodies of *A. bitorquis* and *A. bisporus*....31
Figure S11. Clustering analysis of orthologous genes unique to *A. bitorquis* BH01....32
References....33

Table S1. Statistics of Oxford Nanopore PromethION sequencing data volume of *A. bitorquis* BH01 genome.

Rank	Flag	TotalBase	TotalReads	MaxLen	AvgLen	N50	L50	N90	L90	meanQ
>0	all	10,118,361,858	1,186,113	206,870	8,530.68	20,699	150,958	3,503	627,256	11.45
>0	pass	9,826,399,018	1,144,598	184,094	8,585.02	20,781	146,234	3,520	606,091	11.64
>0	fail	291,962,840	41,515	206,870	7,032.7	17,739	4,789	3,012	21,341	6.23
>5000	all	8,515,574,252	485,578	206,870	17,536.98	24,808	115,620	7,933	349,790	11.52
>5000	pass	8,279,606,619	471,207	184,094	17,571.06	24,871	112,238	7,948	339,301	11.68
>5000	fail	235,967,633	14,371	206,870	16,419.7	22,814	3,402	7,500	10,502	6.24
>10000	all	7,190,712,767	296,637	206,870	24,240.78	28,286	90,616	13,504	234,818	11.51
>10000	pass	6,996,745,125	288,263	184,094	24,272.08	28,339	88,078	13,519	228,156	11.66
>10000	fail	193,967,642	8,374	206,870	23,163.08	26,550	2,550	13,040	6,666	6.24
>50000	all	765,467,143	12,969	206,870	59,022.83	57,413	5,787	51,223	11,457	11.54
>50000	pass	746,612,565	12,659	184,094	58,978.79	57,395	5,652	51,218	11,184	11.67
>50000	fail	18,854,578	310	206,870	60,821.21	58,178	135	51,487	273	6.25
>100000	all	8,722,701	76	206,870	114,772.38	110,818	34	100,681	68	9.51
>100000	pass	7,935,511	70	184,094	113,364.44	110,818	32	100,533	63	9.89
>100000	fail	787,190	6	206,870	131,198.33	110,326	3	102,390	6	5.08

Rank is the gradient of data length, **>0** is that all data; **Flag** is data type, **all** is all sequencing data, **pass** is effective sequencing data, **fail** is filtration data; **TotalBase** is the number of base; **TotalReads** is the number of reads; **MaxLen** is maximum length of data; **AvgLen** is average length of data; **N50** is N50 of data, all reads are summed in order from long to short, and when additive length up to half of all reads total length, the last read length added is N50; **L50** is L50 of data, all reads are accumulated in turn ranking in order from long to short, when additive length up to half of all reads total length, the number of sequences is L50; **N90** is N90 of data, algorithm the same as N50; **L90** is L90 of data, algorithm the same asL50; **meanQ** is mean quality value.

Table S2. Statistics of Illumina NovaSeq sequencing data volume information of *A. bitorquis* BH01 genome.

Sample_name	Total_reads	Total_bases	GC_content	Q20	Q30
rawdata	34,235,978	5,135,396,700	45.19%	97.71%	93.35%
cleandata	34,235,978	5,135,396,700	45.19%	97.71%	93.35%

Sample name is data type; **Total_reads** is sequencing reads number; **Total_bases** is total sequencing base number; **GC_Content** is G/C base number as a percentage of total base number; **Q20**、**Q30** respectively are Phred value greater than 20、30 base as a percentage of total base.

Table S3. Assembly results statistics of *A. bitorquis* BH01 genome.

Item	Value
Total_length(bp)	32,345,193
Total_length_withoutN(bp)	32,345,193
Total_number	22
GC_content(%)	46.05
N50(bp)	2,052,213
N90(bp)	718,652
Average(bp)	1,470,236.05
Median(bp)	1,485,428.50
Min(bp)	113,555
Max(bp)	3,210,751

Total_length is the length of the assembly; **Total_length_withoutN** is the length of the assembly without gaps; **Total_number** is the number of sequences assembled; **GC_content** is the GC content; **N50** is the N50 of the data, all sequences are sorted from longest to shortest and then added in order, when the summed length reaches half of the total length, the last added sequence length is N50; **N90** is the same as N50; **Average** is the average length; **Median** is the median length; **Min** is the minimum length; **Max** is the maximum length.

Table S4. Genome size estimates for *A. bitorquis* BH01 genome.

Item	Number
Heterozygosity	0.665%
Genome Haploid Length	33,542,299 bp
Genome Repeat Length	10,787,297 bp
Genome Unique Length	22,755,002 bp
Model Fit	97.543%
Read Error Rate	0.049%

Table S5. Statistics of Illumina NovaSeq sequencing data mapping of *A. bitorquis* BH01 genome.

Type	Number
map_rate	92.19%
Average_depth	144.95
Coverage	99.83%

map_rate is Illumina NovaSeq sequencing data mapping rates; **Average_depth** is average coverage depth; **Coverage** is coverage rate.

Table S6. Statistics of BUSCO evaluation of *A. bitorquis* BH01 genome.

Item	Number	Percent (%)
Complete BUSCOs (C)	688	90.8
Complete and single-copy BUSCOs (S)	678	89.4
Complete and duplicated BUSCOs (D)	10	1.3
Fragmented BUSCOs (F)	14	1.8
Missing BUSCOs (M)	56	7.4
Total BUSCO groups searched	758	100.0

Table S7. Annotation Statistics of coding gene from the *A. bitorquis* BH01 genome.

Type	Number
Total number of gene	10,028
Average of mRNA length	1,883.52
Average of cds length	1,389.56
Average of exon number	7.71
Average of exon length	180.16
Average of intron length	73.58
Total number of exon	77,347
Total number of intron	67,319
Total intron length	4,953,275

The total number of gene is the total number of genes; **the average of mRNA_length** is the average length of mRNA; **the average of cds_length** is the average length of CDS; **the average of exon_number** is the average number of exons per gene. **the average of exon_length** is the average exon length; **the average of intron_length** is the average intron length; **the total number of exon** is the total number of exons; **the total number of intron** is the total number of introns; **the total intron length** is the total intron length.

Table S8. Statistics of non-coding RNA annotation results in *A. bitorquis* BH01 genome.

Class	number	totalLen(bp)	meanLen(bp)
rRNA	25	35,291	1,411
sRNA	1	341	341
snRNA	11	1,408	128
tRNA	109	9,205	84

rRNA is ribosomal RNA; **tRNA** is transport RNA; **sRNA** is small regulatory RNA; **snRNA** is nucleolar small RNA. **totalLen** and **meanLen** are the total length and mean length.

Table S9. Statistics of *A. bitorquis* BH01 repetitive sequence annotation results.

Item	Subfamily	Number	Length(bp)	Coverage
SINE	/	6	386	0.00%
LINE	/	1,041	1,530,220	4.73%
LTR	/	2,613	3,934,278	12.16%
LTR	Gypsy	1,379	2,495,454	7.72%
LTR	Copia	1,158	1,434,206	4.43%
DNA	/	355	104,334	0.32%
Satellite	/	9	719	0.00%
Simple_repeat	/	3,378	127,786	0.40%
Low_complexity	/	624	30,758	0.10%
Other	/	33	2,719	0.01%
Unknown	/	3,679	2,046,343	6.33%
Total	/	11,738	7,694,206	23.79%

SINE is short scattered element; **LINE** is Long scattered element; **LTR** is long terminal repetition, mainly include two types, Gypsy and Copia; **DNA** is transposons; **Satellite** is satellite repetitive sequence; **Low_complexity** is Low_complexity repetition; **Other** is other types repetition; **Unknown** is unknown repetitive sequence; **Total** is total repetitive sequence.

Table S10. Statistics of *A. bitorquis* BH01 protein-coding gene annotation.

Item	Count	Percentage
All	10,028	100%
Annotation	9,253	92.27%
Uniprot	5,058	50.44%
Pfam	7,023	70.03%
Refseq	3,484	34.74%
Nr	9,216	91.90%
Interproscan	6,991	69.71%
GO	5,021	50.07%
KEGG	3,816	38.05%
Pathway	2,304	22.98%
COG	976	9.73%

Annotation is the gene with at least one annotation; Uniprot is the gene annotated to the Uniprot database; Pfam is the gene that is annotated to the Pfam database; Refseq is the gene that is annotated to the Refseq database; Nr is the gene that is annotated to the Nr database; Interproscan is the gene that is annotated to the Interproscan GO is the gene annotated to the GO database; KEGG is the gene that is annotated to the KEGG database; Pathway is the gene that is annotated to the KEGG Pathway database; COG is the gene that is annotated to the COG database.

Table S11. The composition of CAZymes of *Agaricus* and edible mushrooms.

Species	GT	CBM	PL	CE	AA	GH
Agaricus bisporus BH01	4	9	8	14	22	66
Agaricus bisporus var *burnettii* H119	6	8	7	14	32	69
*Oudemansiella_raphanipes*_CGG-A-s2	8	7	21	26	89	153
Pleurotus ostreatus PC9	2	20	20	19	83	121
*Lyophyllum_decastes*_LRG-d1-5	5	17	17	21	64	114
*Stropharia_rugosoannulata*_A15	2	11	6	29	80	105
*Pleurotus_giganteus*_zhudugu2	4	15	11	14	81	107
Lentinula edodes Lenedo1	2	10	8	15	40	131
Morchella sextelata SCLS	4	4	23	23	50	100
Cyclocybe aegerita AAE3	2	11	10	17	63	89
Armillaria mellea ELDO17	1	11	15	25	48	90
Hericium coralloides FP-101451	1	5	3	16	34	87
Lactarius deliciosus EDB83	6	3	5	7	47	64
Paxillus involutus ATCC 200175	4	3	5	8	29	63
Cordyceps militaris CM01	3	3	3	6	22	71
Boletus edulis BED1	3	1	1	7	19	74
Grifola frondosa 9006-11	2	2	3	5	37	51
Sparassis crispa SCP_1.1	3	0	5	4	15	58

Table S12. GC contents of the mitogenomes of *A. bisporus* var. *bisporus* H97 and *A. bitorquis* BH01.

**Gene**	***Agaricus bisporus* var. *bisporus* H97**	***Agaricus bitorquis* BH01**
atp6	27.17%	27.05%
atp8	20.13%	20.13%
atp9	37.39%	36.49%
cob	31.30%	31.64%
cox1	34.66%	34.85%
cox2	31.40%	31.66%
cox3	34.31%	33.83%
nad1	29.61%	29.80%
nad2	24.35%	24.33%
nad3	29.17%	29.17%
nad4	28.19%	28.04%
nad4L	23.22%	23.22%
nad5	29.07%	29.03%
nad6	25.65%	25.65%
rps3	23.13%	23.13%

Table S13. Analysis of orthologous genes unique to *A. bitorquis* BH01.

Protein ID	Identify	E-Value	function	Accession
g7821.t1				
g7821.t2				
g7839.t1	34.56%	3e-62	kinase-like protein	KAF9447093.1
g7840.t1	37.22%	5e-133	kinase-like protein	KAF9444021.1
g7841.t1	31.33%	3e-63	Mitogen-activated protein kinase YODA	KXN91139.1
g7847.t1	96.43%	3e-28	hypothetical protein	EKV48620.1
g7852.t1	37.22%	2e-131	kinase-like protein	KAF9444021.1
g7853.t1	30.95%	4e-63	Mitogen-activated protein kinase YODA	KXN91139.1
g7853.t2	28.73%	4e-46	Mitogen-activated protein kinase YODA	KXN91139.1
g7859.t1	96.43%	3e-28	hypothetical protein	EKV48620.1
g7864.t1	58.57%	2e-61	hypothetical protein	EKV48615.1
g7864.t2	37.70%	8e-56	Mitochondrial substrate carrier family protein S	KXN81264.1
g7896.t1	82.18%	0.0	hypothetical protein	KAF7782841.1
g7902.t1	82.65%	9e-171	hypothetical protein	KAF7782841.1
g7979.t1	75.12%	8e-106	hypothetical protein	KAF7783187.1
g7979.t2	72.46%	2e-135	hypothetical protein	KAF7783187.1
g8332.t1	95.42%	6e-167	hypothetical protein	KAF7782468.1
g8332.t2	93.09%	2e-167	hypothetical protein	KAF7782468.1
g8413.t1				
g8413.t2	46.94%	0.022	Git3 domain-containing protein	KAF7365684.1
g8494.t1	47.52%	5e-17	transcriptional regulator family	KAF7782328.1
g8494.t2	38.55%	1e-20	transcriptional regulator family	KAF7782328.1
g8550.t1	47.98%	1e-35	5',5'''-P-1, P-4-tetraphosphate phosphorylase 2	KXN91000.1
g8550.t2	48.26%	2e-37	5',5'''-P-1, P-4-tetraphosphate phosphorylase 2	KXN91000.1
g8633.t1	46.10%	9e-74	NAD(P)-binding protein	KAF9447064.1
g8633.t2	46.29%	2e-72	NAD(P)-binding protein	KAF9447064.1
g8676.t1				
g8676.t2				
g8738.t1				
g8738.t2				
g8786.t1	43.37%	4e-08	hypothetical protein	EKV41984.1
g8786.t2				
g8786.t3	46.61%	1e-10	hypothetical protein	EKV41986.1
g8787.t1	43.97%	2e-17	hypothetical protein	KAF7782961.1
g8787.t2	43.97%	2e-16	hypothetical protein	KAF7782961.1
g3916.t1				
g3947.t1	40.79%	0.044	high mobility group protein HMGI-C	KAG7320396.1
g3947.t2	42.11%	0.008	high mobility group	KAG7320396.1

			protein HMGI-C	
g4955.t1	90.04%	2e-174	hypothetical protein	EKV50900.1
g4955.t2	90.38%	3e-174	hypothetical protein	EKV50900.1
g4988.t1				
g4988.t2				
g5012.t1				
g5012.t2				
g5041.t1	60.37%	4e-56	hypothetical protein	KAF7763932.1
g5044.t1	61.33%	3e-22	retrovirus-related pol polyprotein	EDR06651.1
g5076.t1	90.22%	1e-47	transcriptional regulator family: GATA type zinc finger	KAF7775615.1
g5076.t2	91.21%	2e-49	transcriptional regulator family: GATA type zinc finger	KAF7775615.1
g5122.t1				
g5122.t2				
g5170.t1	72.93%	5e-50	Cullin	TRM67470.1
g5177.t1	74.24%	4e-53	ubiquitin-protein ligase	KAH6902807.1
g5862.t1				
g6015.t1	68.35%	4e-24	hypothetical protein	EKM78018.1
g6085.t1	54.00%	3e-75	hypothetical protein	EKM76077.1
g6085.t2	54.80%	2e-76	hypothetical protein	EKM76077.1
g6096.t1				
g6096.t2				
g1480.t1	84.58%	6e-122	hypothetical protein	KAF7768336.1
g1480.t2	81.53%	4e-75	hypothetical protein	KAF7768336.1
g1682.t1	61.82%	3e-12	beta-lactamase/transpeptidase-like protein	KAF9449778.1
g1682.t2	61.82%	5e-12	beta-lactamase/transpeptidase-like protein	KAF9449778.1
g1776.t2	88.02%	1e-119	hypothetical protein	KAF7768115.1
g1776.t3	87.78%	3e-109	hypothetical protein	KAF7768115.1
g1955.t1	37.96%	8e-63	hypothetical protein	EKV45633.1
g1955.t2	38.20%	5e-63	hypothetical protein	EKV45633.1
g1959.t1	73.75%	3e-78	hypothetical protein	EKM79149.1
g1959.t2	71.93%	2e-81	hypothetical protein	EKM79149.1
g7155.t1	79.11%	0.0	phosphoglycerate mutase-like protein	KAF9449257.1
g7155.t2	78.23%	0.0	phosphoglycerate mutase-like protein	KAF9449257.1
g7209.t1	70.24%	1e-28	hypothetical protein	EKV48690.1
g7209.t2	66.67%	1e-38	hypothetical protein	EKV49576.1
g7245.t1	36.09%	6e-51	S-adenosyl-L-methionine-dependent methyltransferase	KAF9445131.1
g7258.t1	36.88%	2e-51	S-adenosyl-L-methionine-dependent methyltransferase	KAF9445131.1
g7344.t1	72.40%	7e-167	CAZyme family AA3	KAF7771600.1
g7344.t2	72.40%	5e-165	CAZyme family AA3	KAF7771600.1

g7427.t1				
g7427.t2				
g7440.t1	50.71%	3e-113	beta-lactamase-like protein	TFK40308.1
g7440.t2	50.86%	3e-115	beta-lactamase-like protein	TFK40308.1
g7688.t1	70.80%	2e-113	PIN domain-like protein	KAF9451879.1
g7688.t2	70.40%	1e-112	PIN domain-like protein	KAF9451879.1
g7733.t1				
g7735.t1				
g7736.t1	76.92%	4e-33	hypothetical protein	EKV48961.1
g7740.t1	47.46%	0.039	hypothetical protein	KAF8239668.1
g2129.t1				
g2130.t1	73.13%	0.0	HCO_3 transporter family-domain-containing protein	KAF8158230.1
g2135.t1	30.47%	2e-33	Hypothetical protein	KAJ3566546.1
g2136.t1	79.58%	1e-99	Hypothetical protein	KAF7763888.1
g2138.t1				
g2145.t1	84.21%	3e-106	Hypothetical protein	KAF7763888.1
g2147.t1	46.75%	5e-08	Hypothetical protein	KAF5382182.1
g2150.t1				
g2151.t1	86.76%	0.0	Hypothetical protein	KAF7762649.1
g2251.t1	42.51%	1e-37	Hypothetical protein	KAF5348801.1
g2251.t2	41.09%	6e-35	Hypothetical protein	KAF5348801.1
g2322.t1	84.67%	0.0	Hypothetical protein	KAF7762180.1
g2322.t2	85.46%	0.0	Hypothetical protein	KAF7762180.1
g2333.t1	89.30%	8e-116	Hypothetical protein	KAF7762169.1
g2333.t2	87.43%	1e-113	hypothetical protein	KAF7762169.1
g2360.t1				
g2360.t2				
g2447.t1				
g2447.t2				
g6310.t1	81.43%	7e-126	hypothetical protein	KAF7770709.1
g6312.t1	79.31%	6e-101	hypothetical protein	KAF7770709.1
g6313.t1				
g6604.t1	55.24%	3e-70	transcriptional regulator family	KAF7770379.1
g6604.t2	51.14%	2e-77	transcriptional regulator family	KAF7770379.1
g6693.t1	66.89%	6e-134	ZIP-like iron-zinc transporter	KAF8817477.1
g6693.t2	66.56%	7e-131	ZIP-like iron-zinc transporter	KAF8817477.1
g6699.t1				
g6699.t2				
g6699.t3				
g3341.t1				
g3341.t2				
g3342.t1				
g3342.t2				
g3344.t1				
g3344.t2				
g3344.t3				
g3344.t4				

g3439.t1	67.77%	1e-113	CAZyme family AA3	KAF7771600.1
g3439.t2	71.73%	5e-153	CAZyme family AA3	KAF7771600.1
g3489.t1	60.00%	4e-17	Alpha/Beta hydrolase protein	TFK42369.1
g3489.t2	64.63%	3e-29	Alpha/Beta hydrolase protein	KAF8163516.1
g3691.t1				
g3691.t2				
g3906.t1	29.91%	1e-04	intersectin-1 isoform X1	KAF0758251.1
g2548.t1	76.01%	0.0	hypothetical protein	KAF7759812.1
g2548.t2	75.77%	0.0	hypothetical protein	KAF7759812.1
g2558.t1	84.85%	2e-28	hypothetical protein	KAF7763439.1
g2558.t2	63.44%	2e-28	hypothetical protein	KAF7763439.1
g2568.t1	77.46%	2e-136	glycoside hydrolase family 27 protein	KAF9448138.1
g2568.t2	77.46%	2e-136	glycoside hydrolase family 27 protein	KAF9448138.1
g2577.t1				
g2577.t2				
g2583.t1				
g2583.t2				
g2618.t1	66.90%	2e-46	hypothetical protein	KAF7763457.1
g2618.t2	61.54%	1e-59	hypothetical protein	KAF7763457.1
g2629.t1				
g2629.t2				
g2645.t1				
g2645.t2				
g2669.t1				
g2669.t2				
g2670.t1				
g2670.t2				
g2672.t1	66.26%	1e-60	hypothetical protein	KAF7763496.1
g2672.t2	52.80%	3e-56	hypothetical protein	KAF7763496.1
g2672.t3	60.44%	3e-69	hypothetical protein	KAF7763496.1
g2826.t1	37.78%	0.003	other/BUB protein kinase	KAF8068848.1
g2826.t2	37.78%	0.004	other/BUB protein kinase	KAF8068848.1
g2826.t3	37.78%	0.005	other/BUB protein kinase	KAF8068848.1
g2830.t1				
g2830.t2				
g2947.t1	40.16%	8e-20	hypothetical protein	KAF7764071.1
g2948.t1	42.47%	3e-09	hypothetical protein	KAF3936583.1
g2950.t1				
g2950.t2				
g501.t1	50.31%	2e-46	hypothetical protein	KAG6815639.1
g562.t1				
g562.t2				
g572.t1	54.29%	6e-49	hypothetical protein	KAF7785220.1
g572.t2	54.29%	5e-48	hypothetical protein	KAF7785220.1
g572.t3	54.29%	6e-50	hypothetical protein	KAF7785220.1
g574.t1	54.14%	1e-42	hypothetical protein	KAF7785220.1
g574.t2	54.67%	7e-41	hypothetical protein	KAF7785220.1
g681.t1	35.28%	1e-42	hypothetical protein	KAF9449339.1

g681.t2	34.97%	9e-42	hypothetical protein	KAF9449339.1
g692.t1				
g698.t1				
g698.t2				
g738.t1	62.70%	3e-112	carbohydrate esterase family 16 protein	KIM66770.1
g738.t2	62.55%	4e-114	carbohydrate esterase family 16 protein	KIM66770.1
g790.t1	58.22%	9e-79	hypothetical protein	KAF7761252.1
g790.t2	59.60%	9e-92	hypothetical protein	KAF7761252.1
g796.t1	40.00%	2e-04	hypothetical protein	KAF7768249.1
g802.t1				
g802.t2				
g947.t1				
g947.t2				
g949.t1	56.82%	5e-05	FAD-binding domain-containing protein	PWN88759.1
g949.t2	51.06%	0.011	putative oxygen-dependent FAD-linked oxidoreductase family protein	GLB39903.1
g1008.t1	76.92%	3e-26	CAZyme family AA3	KAF7777703.1
g1008.t2	76.92%	2e-26	CAZyme family AA3	KAF7777703.1
g1011.t1	71.79%	1e-27	RNI-like protein	KAF9447166.1
g1011.t2	71.79%	1e-27	RNI-like protein	KAF9447166.1
g1068.t1	62.25%	3e-76	Sterol 3-beta-glucosyltransferase UGT80A2	KXN80622.1
g1068.t2	62.25%	1e-77	Sterol 3-beta-glucosyltransferase UGT80A2	KXN80622.1
g1140.t1	70.72%	6e-100	hypothetical protein	KAF7777596.1
g1140.t2	68.18%	8e-71	hypothetical protein	KAF7777596.1
g1318.t1	37.93%	8e-08	hypothetical protein	OGO81950.1
g8979.t1	67.35%	1e-12	phosphatidylethanolamine N-methyltransferase	KAF8816890.1
g8979.t2	56.16%	2e-14	phosphatidylethanolamine N-methyltransferase	KAF8816890.1
g8979.t3	55.45%	2e-24	phosphatidylethanolamine N-methyltransferase	KAF8816890.1
g8995.t1				
g8995.t2				
g9034.t1	52.19%	2e-101	cytochrome P450	EKV43158.1
g9034.t2	52.19%	2e-101	cytochrome P450	EKV43158.1
g9194.t1				
g9194.t2				
g9214.t2	66.01%	0.0	alcohol dehydrogenase	KAF9451722.1
g9217.t2	66.21%	0.0	alcohol dehydrogenase	KAF9451722.1
g6803.t1	32.30%	1e-14	hypothetical protein	KAF7761807.1
g6804.t1	31.84%	7e-17	hypothetical protein	KAF7760454.1
g6812.t1	60.61%	9e-04	hypothetical protein	EKM76409.1
g6812.t2	66.67%	0.002	hypothetical protein	EKM76409.1
g6936.t1				

g6936.t2				
g6995.t1	71.88%	0.013	hypothetical protein	KAG5730867.1
g6995.t2	71.88%	0.013	hypothetical protein	KAG5730867.1
g6996.t1				
g6997.t1				
g6998.t1				
g7071.t1				
g7071.t2				
g7121.t1				
g7129.t1	79.11%	5e-78	hypothetical protein	KAF7761547.1
g7130.t2	61.11%	0.011	hypothetical protein	KAF7761548.1
g7139.t1	66.41%	1e-119	acetoin reductase family protein	KAF9447564.1
g7148.t1	63.88%	6e-132	*O*-methylsterigmatocystin oxidoreductase	KXN81086.1
g7148.t2	53.81%	7e-139	*O*-methylsterigmatocystin oxidoreductase	KXN81086.1
g7148.t3	59.20%	1e-112	*O*-methylsterigmatocystin oxidoreductase	KXN81086.1
g51.t1				
g51.t2				
g87.t1	71.83%	3e-29	hypothetical protein	KAF7759616.1
g184.t1	73.91%	6e-28	hypothetical protein	KAF7759616.1
g189.t1	81.31%	1e-104	hypothetical protein	KAF7759931.1
g189.t2	82.32%	2e-108	hypothetical protein	KAF7759931.1
g6110.t1				
g6110.t2	43.02%	3e-13	hypothetical protein	KAF7760353.1
g6153.t1				
g6153.t2				
g6153.t3				
g6236.t1	90.13%	2e-87	B-(1-6) glucan synthase	EKV48912.1
g6236.t2	90.13%	2e-87	B-(1-6) glucan synthase	EKV48912.1
g6281.t1				
g6286.t1	69.34%	2e-54	hypothetical protein	KAF7773209.1
g6287.t1				
g6289.t1				
g3123.t1				
g3132.t1	81.03%	2e-104	hypothetical protein	KAF7761547.1
g3133.t2	45%	0.011	hypothetical protein	KAF7761548.1
g3142.t1	66.41%	1e-119	acetoin reductase family protein	KAF9447564.1
g3023.t1	75.36%	3e-28	RAD26-like SNF2 family DNA-dependent ATPase	KAF8346616.1
g3023.t2	75.36%	3e-28	RAD26-like SNF2 family DNA-dependent ATPase	KAF8346616.1
g3057.t1	97.26%	2e-38	salt tolerance down-regulator-domain-containing protein	KAF8970654.1
g3057.t2	97.26%	2e-38	salt tolerance down-regulator-domain-containing protein	KAF8970654.1
g3069.t1	69.47%	8e-32	CAZyme family CE10	KAF7759817.1
g3069.t2	69.47%	6e-32	CAZyme family CE10	KAF7759817.1

g3113.t1	59.17%	5e-68	hypothetical protein	KAF4610720.1
g1337.t1	58.52%	0.0	hypothetical protein	KAF7783396.1
g1337.t2	57.50%	0.0	hypothetical protein	KAF7783396.1
g1346.t1	68.31%	0.0	hypothetical protein	KAF7783396.1
g1346.t2	68.07%	0.0	hypothetical protein	KAF7783396.1
g1381.t1	68.50%	0.0	hypothetical protein	KAF7785221.1
g1381.t2	68.99%	0.0	hypothetical protein	KAF7785221.1
g8832.t1				
g8832.t2				
g8851.t1				
g8851.t2				
g8851.t3				
g8853.t1	82.99%	9e-81	hypothetical protein	EKM84331.1
g8861.t1				
g8861.t2				
g8861.t3				
g1.t1	86.89%	2e-28	hypothetical protein	KAF7761647.1
g1.t2	88.06%	9e-33	hypothetical protein	KAF7761647.1
g5.t1				
g5.t2				
g10.t1				
g10.t2	46.91%	3e-11	hypothetical protein	KAF7762179.1
g10.t3	53.45%	1e-08	hypothetical protein	KAF7762179.1
g38.t1	60.36%	1e-55	ubiquitin-conjugating enzyme/RWD-like protein	KAJ4494584.1
g38.t2	55.32%	9e-37	ubiquitin-conjugating enzyme/RWD-like protein	KAJ4494584.1

The analysis result of the entry with a blank comment result is "No significant similarity found".

Table S14. Homologous genes of *A. bisporus* fruiting body development related genes in *A. bitorquis* BH01.

Protein ID	Predicted function	E Value	Identities	Accession No.	References
g7269.t1	polyphenoloxidase	0.0	92%	GU936494	[1]
g7229.t1		e^{-160}	53%		
g7210.t1		e^{-141}	33%		
g7210.t1	polyphenoloxidase	0.0^{3}	72%	GU936493	[1]
g7269.t1		e^{-151}	47%		
g7229.t1		e^{-117}	49%		
g6263.t2	urease	0.0	96%	AJ748112	[2]
g6263.t1		0.0	96%		
g8566.t1	ATP-synthase delta-subunit	$4e^{-83}$	96%	CAB04785.1	[3]
g8615.t1	cell division control	$2e^{-086}$	98%	CAB04784.1	[3]
g4300.t1		$3e^{-057}$	67%		
g4096.t1		$6e^{-025}$	41%		
g7779.t1	hypothetical protein	$1e^{-47}$	81%	AGABI2DRAFT_117525	[4]
g7794.t1		$3e^{-45}$	63%		
g1808.t1	hypothetical protein	0.0	86%	AGABI2DRAFT_119457	[4]
g3058.t1		$4e^{-24}$	32%		
g7264.t1		$8e^{-24}$	31%		
g8587.t1	hypothetical protein	e^{-115}	91%	AGABI2DRAFT_117665	[4]
g9004.t1	hypothetical protein	e^{-130}	51%	AGABI2DRAFT_194024	[4]
g674.t1		e^{-125}	87%		
g671.t1		e^{-114}	77%		
g668.t1		e^{-113}	84%		
g553.t1	hypothetical protein	0.0	91%	AGABI2DRAFT_188444	[4]
g8011.t1	hypothetical protein	$1e^{-087}$	89%	AGABI2DRAFT_136361	[4]
g4325.t1		$1e^{-027}$	45%		
g2507.t1	hypothetical protein	$6e^{-040}$	76%	AGABI2DRAFT_138343	[4]
g2600.t1		$7e^{-040}$	66%		
g2508.t1		$1e^{-035}$	71%		

Descriptions | Graphic Summary | Alignments | Taxonomy

Sequences producing significant alignments Download Select columns Show 100

select all *100 sequences selected* GenBank Graphics Distance tree of results MSA Viewer

Description	Scientific Name	Max Score	Total Score	Query Cover	E value	Per. Ident	Acc. Len	Accession
Agaricus bitorquis strain m6 internal transcribed spacer 1, partial sequence; 5.8S ribosomal RNA gene and interna…	Agaricus bitorquis	1260	1260	98%	0.0	99.57%	740	KY401339.1
Agaricus bitorquis voucher ZRL20190040 small subunit ribosomal RNA gene, partial sequence; internal transcribe…	Agaricus bitorquis	1260	1260	98%	0.0	99.57%	752	MW555377.1
Agaricus bitorquis voucher ZRL20190039 internal transcribed spacer 1, partial sequence; 5.8S ribosomal RNA ge…	Agaricus bitorquis	1260	1260	98%	0.0	99.42%	739	MW555376.1
Agaricus bitorquis voucher ZRL20190020 small subunit ribosomal RNA gene, partial sequence; internal transcribe…	Agaricus bitorquis	1260	1260	98%	0.0	99.57%	758	MW555361.1
Agaricus bitorquis strain m7 internal transcribed spacer 1, partial sequence; 5.8S ribosomal RNA gene and interna…	Agaricus bitorquis	1254	1254	98%	0.0	99.42%	736	KY401340.1
Agaricus bitorquis strain a4 internal transcribed spacer 1, partial sequence; 5.8S ribosomal RNA gene and internal…	Agaricus bitorquis	1254	1254	97%	0.0	99.71%	739	KY401335.1
Agaricus bitorquis voucher ZRL20190047 small subunit ribosomal RNA gene, partial sequence; internal transcribe…	Agaricus bitorquis	1254	1254	98%	0.0	99.28%	760	MW555384.1
Agaricus bitorquis isolate EGDA-A1 small subunit ribosomal RNA gene, partial sequence; internal transcribed spa…	Agaricus bitorquis	1254	1254	98%	0.0	99.42%	752	MW915591.1
Agaricus bitorquis strain g1 internal transcribed spacer 1, partial sequence; 5.8S ribosomal RNA gene and internal…	Agaricus bitorquis	1253	1253	98%	0.0	99.42%	740	KY401331.1
Agaricus bitorquis voucher ZRL20190037 small subunit ribosomal RNA gene, partial sequence; internal transcribe…	Agaricus bitorquis	1253	1253	97%	0.0	99.56%	735	MW555374.1
Agaricus bitorquis genomic DNA containing ITS1, 5.8S rRNA gene and ITS2, strain EGW1, isolate 5	Agaricus bitorquis	1251	1251	97%	0.0	99.56%	754	LK024176.1
Agaricus bitorquis strain m14 small subunit ribosomal RNA gene, partial sequence; internal transcribed spacer 1, 5…	Agaricus bitorquis	1249	1249	97%	0.0	99.56%	741	KY401341.1

Figure S1. ITS alignment of the strain BH01.

The ITS of strain BH01 was aligned to the nr database of NCBI.

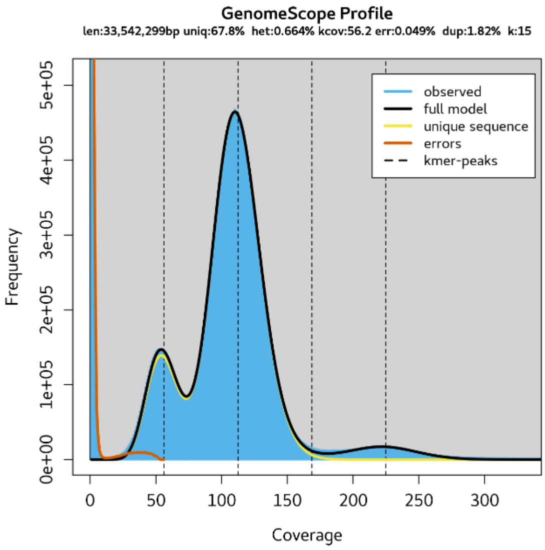

Figure S2. *K*-mer-Depth and *K*-mer Species-Frequency Distribution Plot.

The blue line represents the actual *K*-mer curve, the black line is the k-mer curve estimated by the model, the yellow line is the *K*-mer curve corresponding to the unique data, the red line represents the error curve due to sequencing errors, and the dashed line represents speculation *K*-mer peak.

Using the reads obtained by sequencing, *K*-mer-based analysis was used to estimate the genome size and heterozygosity. A *K*-mer refers to a sequence of K bp in length. Iteratively select a sequence of length *K* bases from a continuous sequence. If the length of the sequence is L and the length of the *K*-mer is K, then L-K+1 *K*-mers can be obtained. We take *K*-mers for the reads obtained by sequencing, and then count the frequency of each *K*-mer. According to the Lander_waterman algorithm, the genome size (G) satisfies the following formula:

$$C_{base} = C_{k-mer} \times \frac{L}{L-K+1}$$

$$G = \frac{n_{k-mer}}{C_{k-mer}} = \frac{n_{base}}{C_{base}}$$

C_{base} and $C_{k\text{-}mer}$ are the expected depth of coverage and *K*-mer, and nbase and n*k*-mer are the total number of bases and the total number of K-mers in the sequence. In the case of a certain amount of data, the depth frequency of *K*-mer is subject to Poisson distribution, so the peak of the *K*-mer depth frequency distribution is the corresponding depth, which is used as an estimate of the expected depth of *K*-mer.

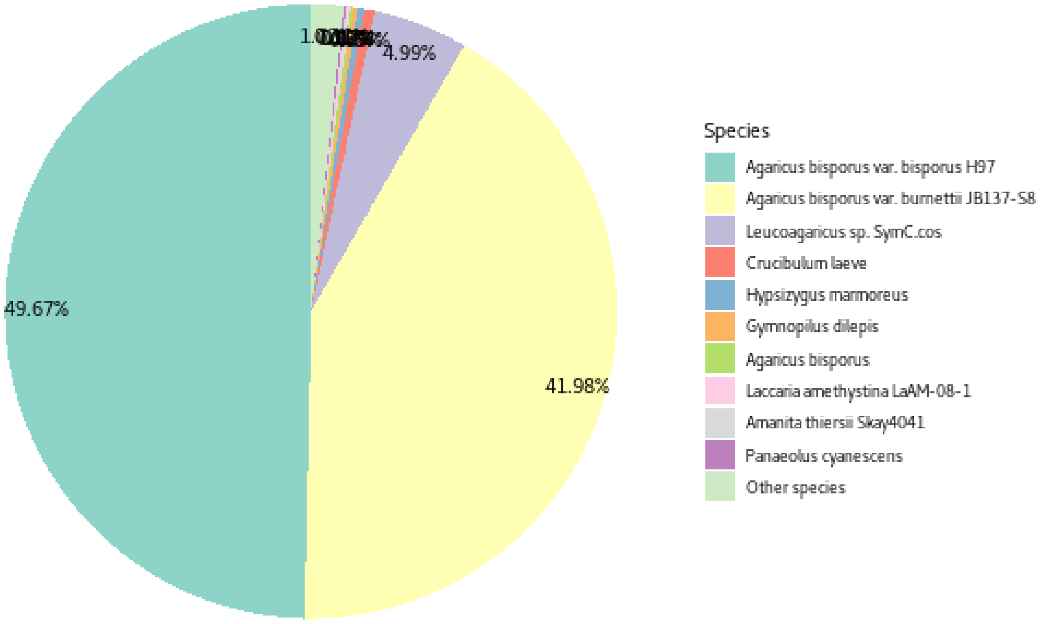

Figure S3. Species distribution map of Nr database alignment to sequences.

According to the results of the Nr library match, the top 10 species were counted and the rest were classified as other species, and the distribution of these species was mapped according to their proportion.

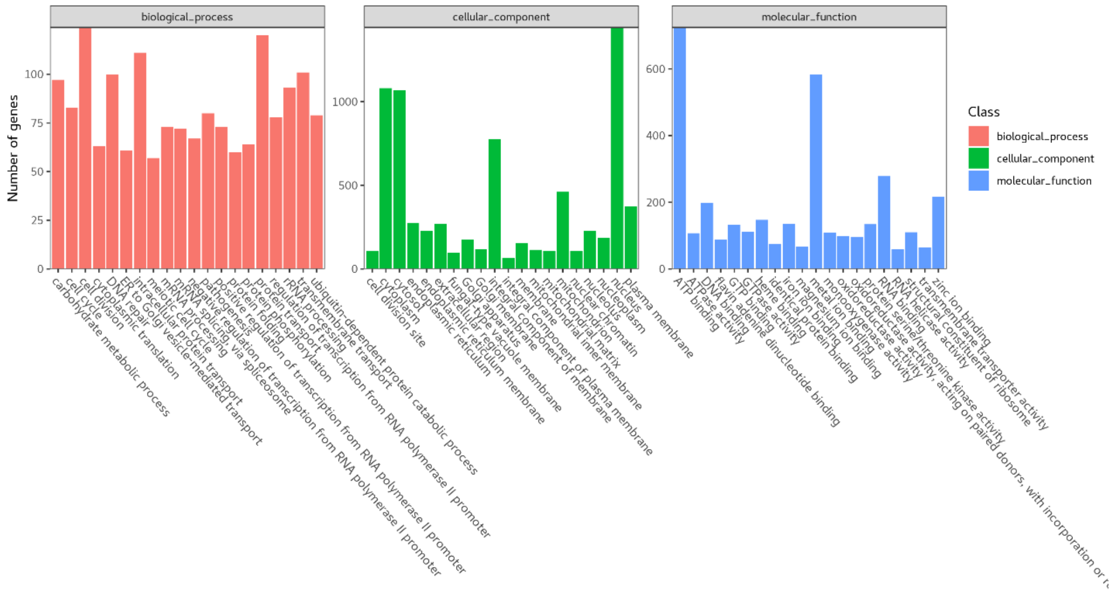

Figure S4. Statistical map of functional annotation classification based on GO database

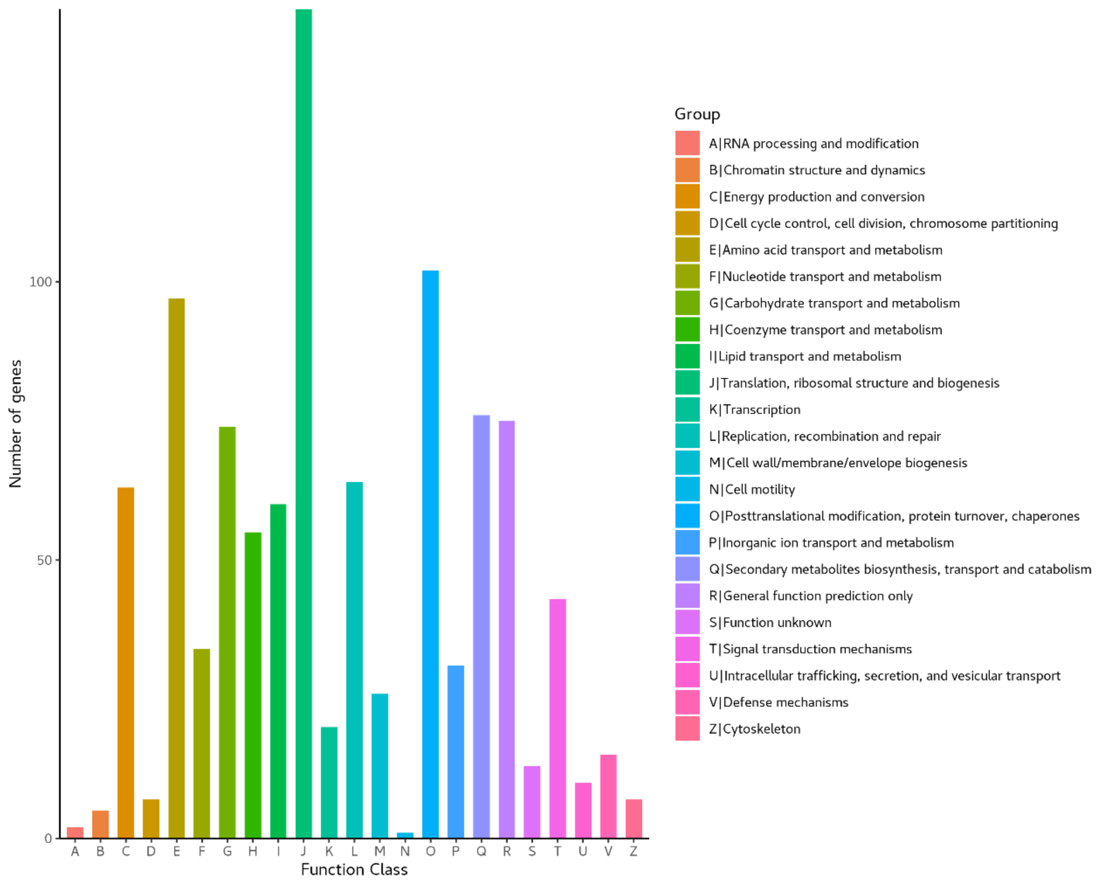

Figure S5. Statistical Chart of COG Functional Annotated Classification.

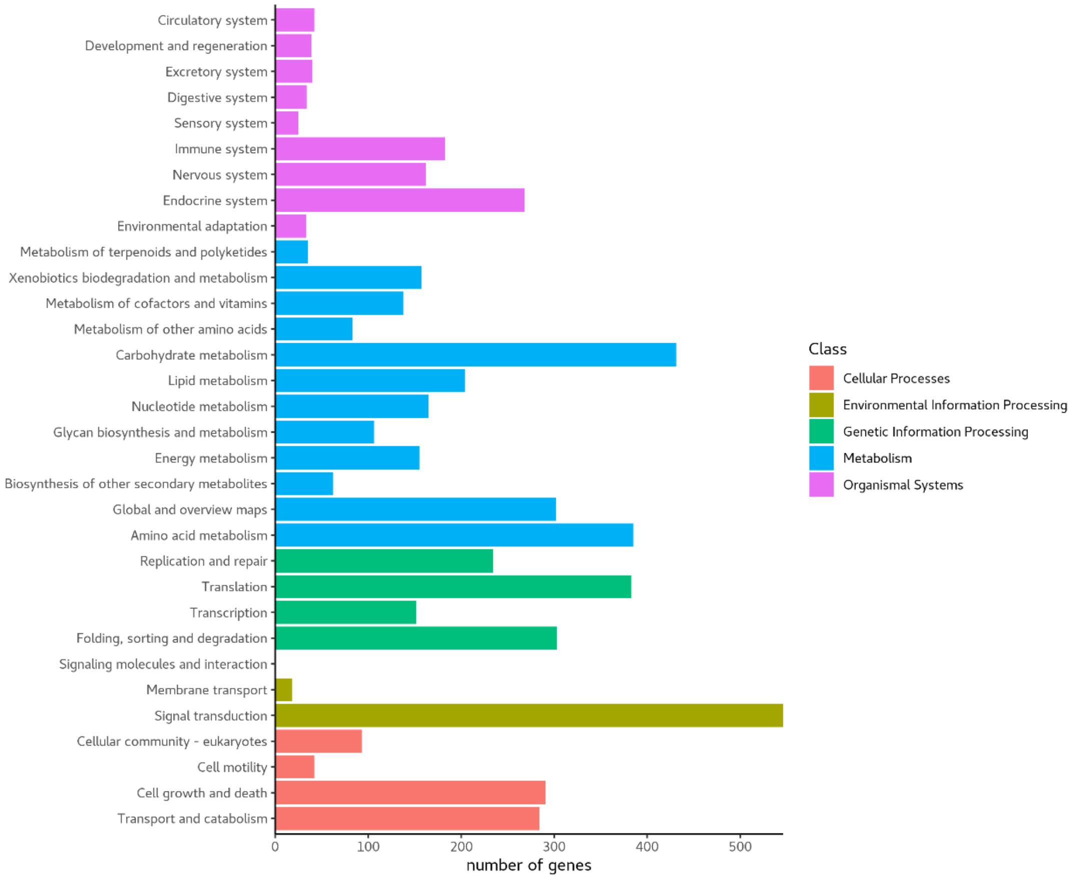

Figure S6. KEGG Pathway Functional Classification Chart.

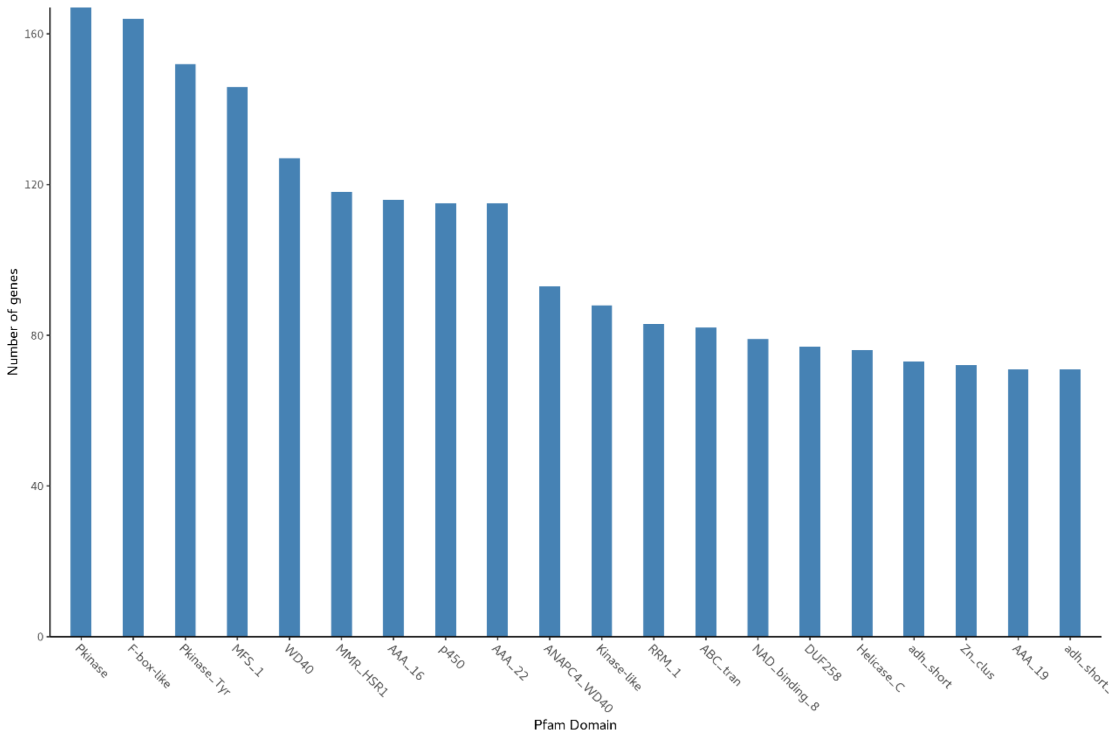

Figure S7. Domain annotation based on the Pfam database.

Statistical summaries of genes annotated for each domain are made and the top 20 most annotated domains are plotted.

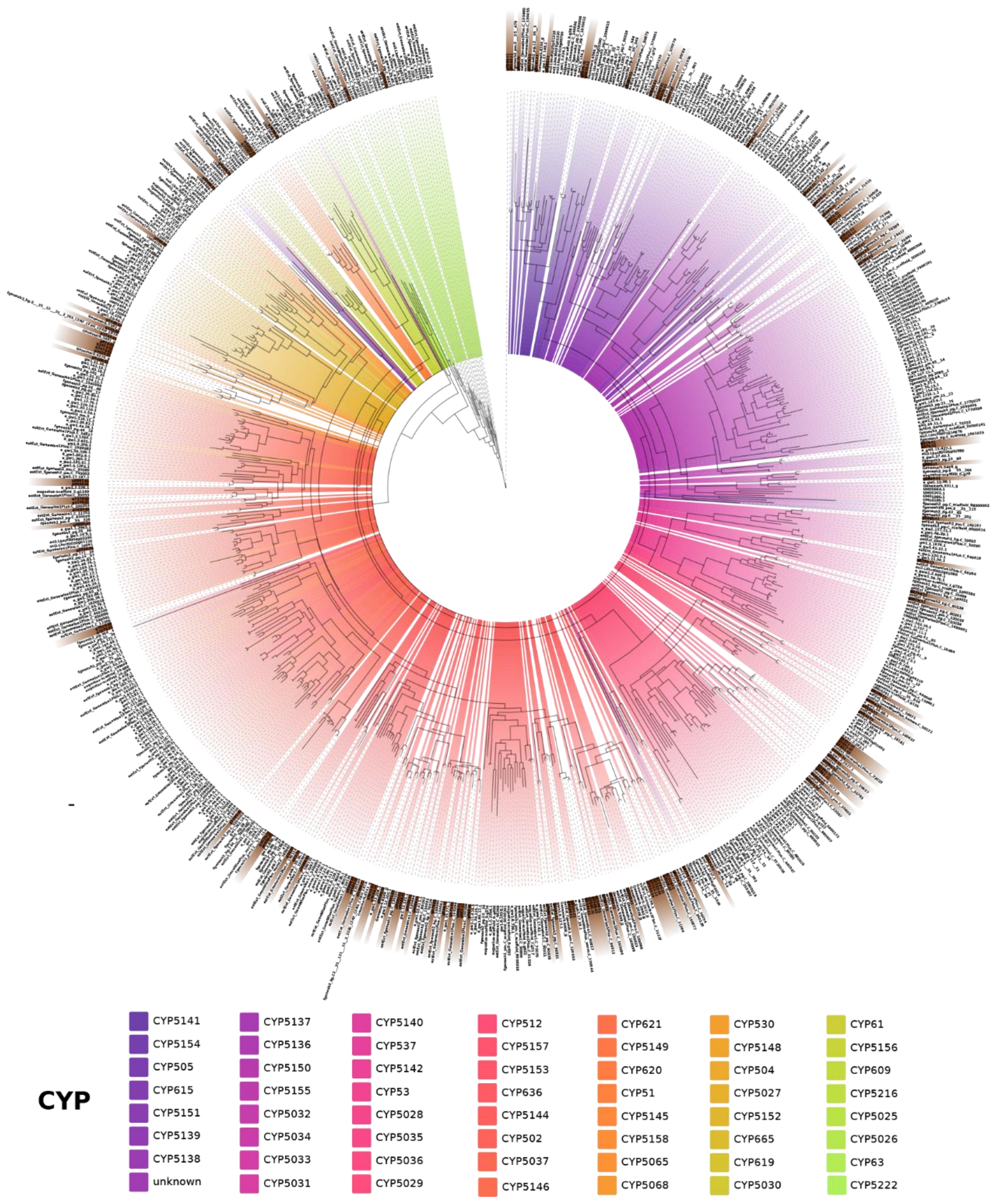

Figure S8. P450s Cluster analysis of *A. bitorquis* BH01 and other Basidiomycetes.

The sequences of the branch with color background and the corresponding sequence name without background are from Fungal Cytochrome P450 Database (http://passport.riceblast.snu.ac.kr/?t=P450). The background color of the branch corresponds to the category to which it belongs. The sequences with dark brown background and corresponding branch without background are P450 sequences from *A. bitorquis* BH01. Multiple sequence pairs are implemented with mafft v7.505 with parameters --maxiterate 1000 --localpair. The evolutionary tree was constructed by IQtree v2.2.3[5] with the parameters -m MFP -bb 1000 -alrt 1000 -abayes -nt AUTO.

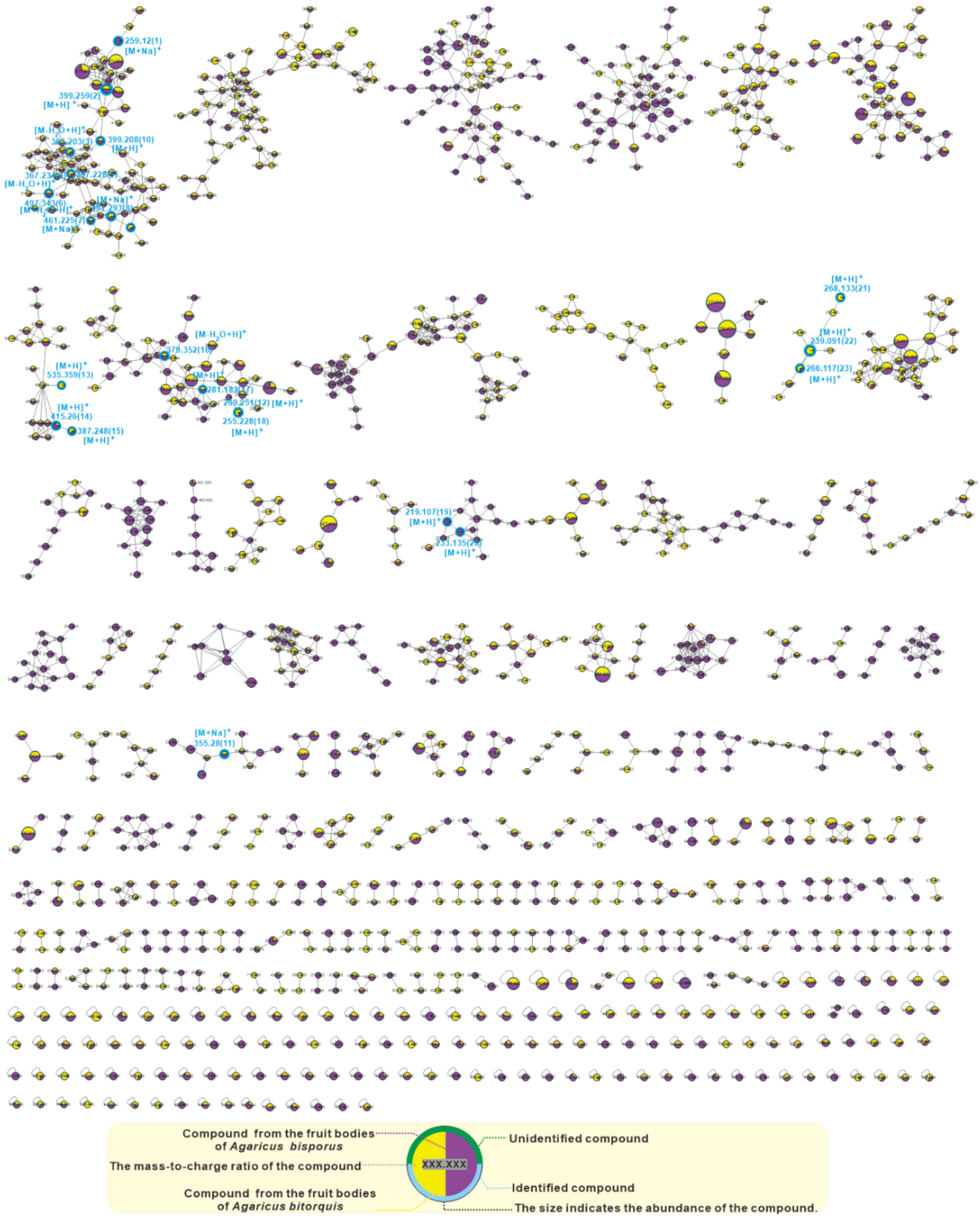

Figure S9. Molecular network analysis of metabolites from the fruiting bodies of *A. bitorquis* and *A. bisporus*.

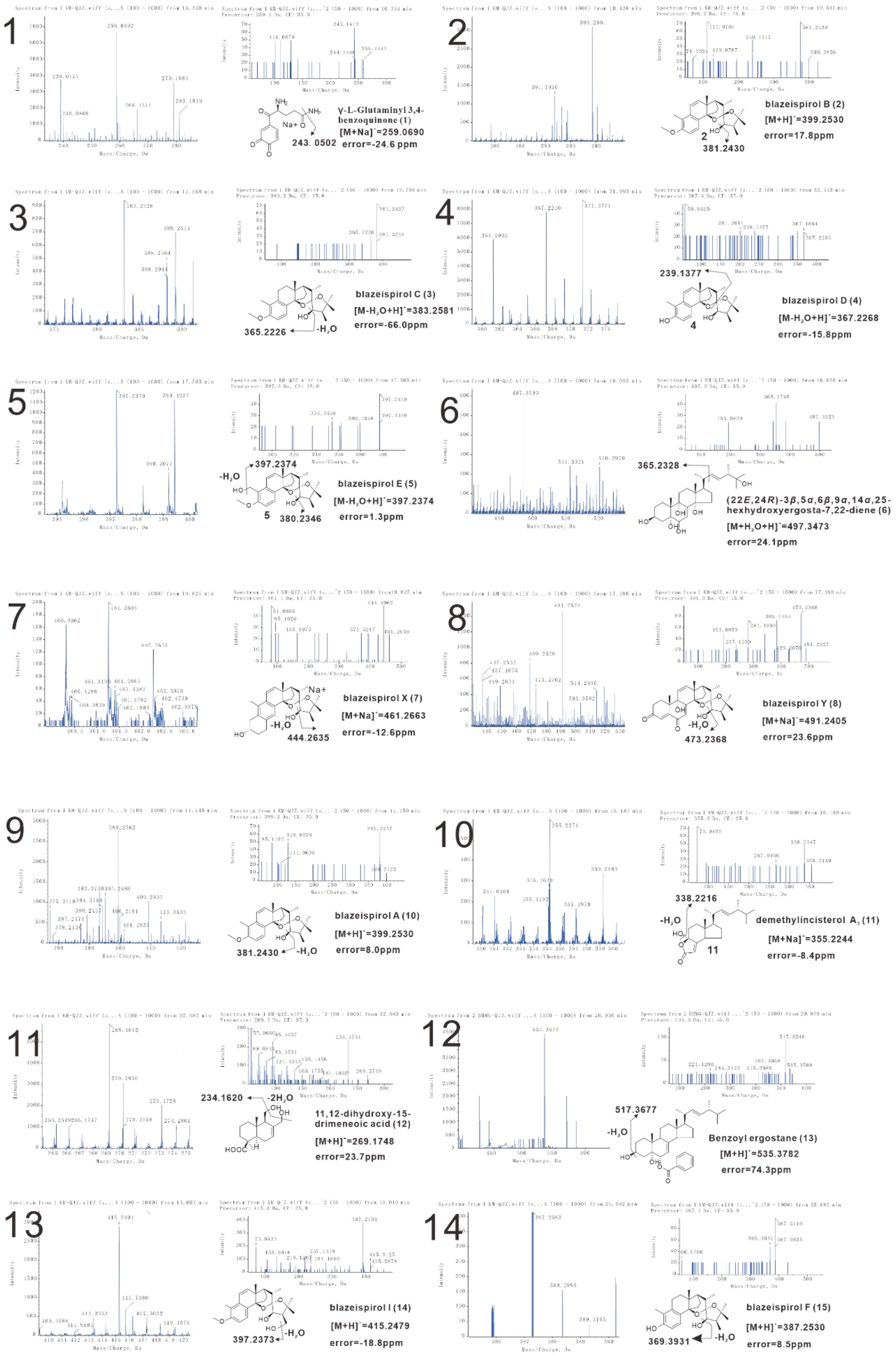

1
γ-L-Glutaminyl 3,4-benzoquinone (1)
[M+Na]+=259.0690
243. 0502
error=-24.6 ppm
2
blazeispirol B (2)
[M+H]+=399.2530
error=17.8ppm
381.2430
3
blazeispirol C (3)
[M-H2O+H]+=383.2581
error=-66.0ppm
365.2226
-H2O
4
239.1377
blazeispirol D (4)
[M-H2O+H]+=367.2268
error=-15.8ppm
5
397.2374
-H2O
blazeispirol E (5)
[M-H2O+H]+=397.2374
error=1.3ppm
380.2346
6
365.2328
(22E,24R)-3β,5α,6β,9α,14α,25-hexhydroxyergosta-7,22-diene (6)
[M+H2O+H]+=497.3473
error=24.1ppm
7
Na+
blazeispirol X (7)
[M+Na]+=461.2663
error=-12.6ppm
-H2O
444.2635
8
blazeispirol Y (8)
[M+Na]+=491.2405
error=23.6ppm
-H2O
473.2368
9
blazeispirol A (10)
[M+H]+=399.2530
error=8.0ppm
381.2430
-H2O
10
338.2216
-H2O
demethylincisterol A3 (11)
[M+Na]+=355.2244
error=-8.4ppm
11
234.1620
-2H2O
11,12-dihydroxy-15-drimeneoic acid (12)
[M+H]+=269.1748
error=23.7ppm
12
517.3677
-H2O
Benzoyl ergostane (13)
[M+H]+=535.3782
error=74.3ppm
13
blazeispirol I (14)
[M+H]+=415.2479
error=-18.8ppm
397.2373
-H2O
14
blazeispirol F (15)
[M+H]+=387.2530
error=8.5ppm
369.3931
-H2O

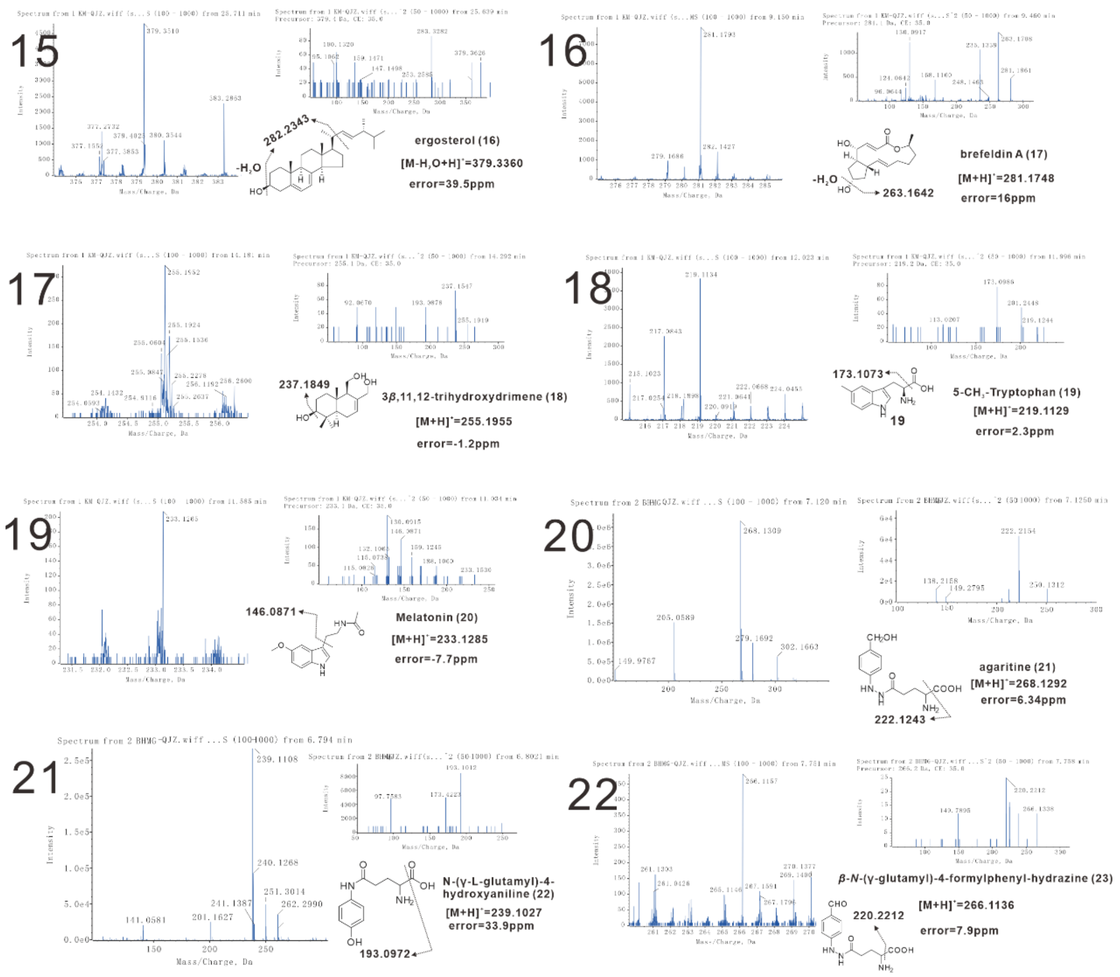

Figure S10. The LC-ESI-HRMS and LC-ESI-HRMS/MS spectrums of isolates from the fruiting bodies of *A. bitorquis* and *A. bisporus*.

Mass spectral data were acquired in positive ion mode.
The spectrum numbers correspond to the compound numbers in the manuscript.

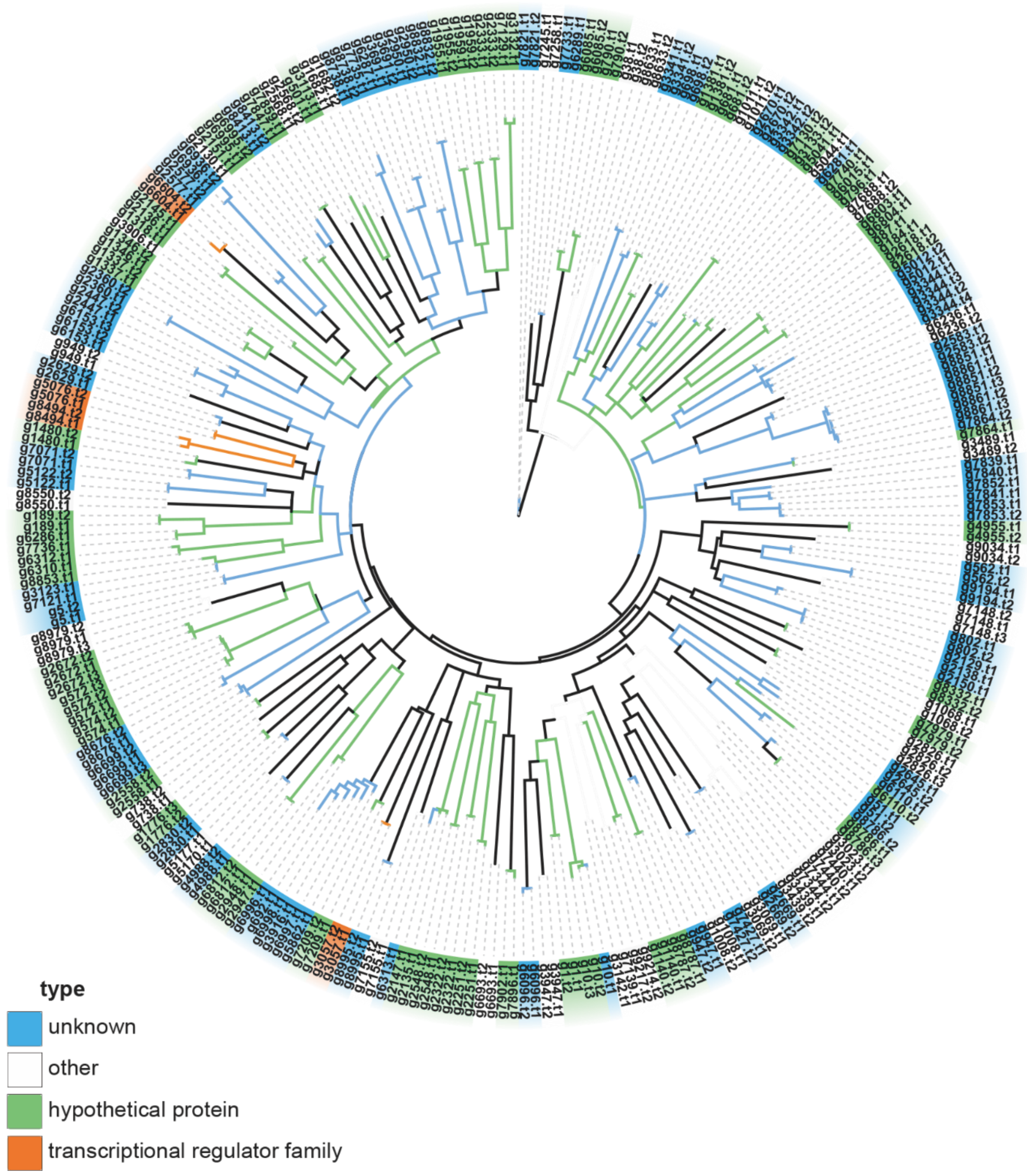

Figure S11. Clustering analysis of orthologous genes unique to *A. bitorquis* BH01.

References

1. Li, N.-y.; Cai, W.-m.; Jin, Q.-l.; Qin, Q.-p.; Ran, F.-l. Molecular Cloning and Expression of Polyphenoloxidase Genes from the Mushroom, Agaricus bisporus. *Agricultural Sciences in China* **2011**, *10*, 185-194, doi:10.1016/S1671-2927(09)60305-9.
2. Wagemaker, M.J.M.; Eastwood, D.C.; van der Drift, C.; Jetten, M.S.M.; Burton, K.; Van Griensven, L.J.L.D.; Op den Camp, H.J.M. Expression of the urease gene of Agaricus bisporus: a tool for studying fruit body formation and post-harvest development. *Applied Microbiology and Biotechnology* **2006**, *71*, 486-492, doi:10.1007/s00253-005-0185-5.
3. De Groot, P.W.J.; Schaap, P.J.; Van Griensven, L.J.L.D.; Visser, J. Isolation of developmentally regulated genes from the edible mushroom Agaricus bisporus. *Microbiology* **1997**, *143*, 1993-2001, doi:10.1099/00221287-143-6-1993.
4. Yuan, S.; Qiuhui, H.; Anxiang, S.; Fei, P.; Gaoxing, M.; Ning, M.; Wenjian, Y. Transcriptomic Analysis of the Mechanism Underlying the Effect of Methyl Jasmonate on the Size of Agaricus bisporus. *Food Science* **2021**, *42*, 130-137, doi:10.7506/spkx1002-6630-20200720-258.
5. Minh, B.Q.; Schmidt, H.A.; Chernomor, O.; Schrempf, D.; Woodhams, M.D.; von Haeseler, A.; Lanfear, R. IQ-TREE 2: New Models and Efficient Methods for Phylogenetic Inference in the Genomic Era. *Molecular Biology and Evolution* **2020**, *37*, 1530-1534, doi:10.1093/molbev/msaa015.
